# Barring the gates to the battleground: DDR1 promotes immune exclusion in solid tumors

**DOI:** 10.1038/s41392-022-00877-4

**Published:** 2022-01-13

**Authors:** Dimitrios L. Wagner, Enrico Klotzsch

**Affiliations:** 1 Berlin Center for Advanced Therapies (BeCAT) and BIH Center for Regenerative Therapies (BCRT), Charité - Universitätsmedizin Berlin, Augustenburger Platz 1, 13353, Berlin, Germany; 2 Institute for Biology, Experimental Biophysics/Mechanobiology, Humboldt University of Berlin, Berlin, Germany; 3 Laboratory of Applied Mechanobiology, Department for Health Sciences and Technology, ETH Zurich, Zurich, Switzerland

**Keywords:** Biophysics, Translational research

In a recent article in *Nature*, Sun et al.^1^ identify a novel mechanism of how solid tumors prevent immune cell infiltration into cancer tissue by collagen-remodeling. Furthermore, they develop and test a new monoclonal antibody that may be used as a stand-alone treatment or allow the optimization of T cell-targeted immunotherapy in the future.

Cancer is one of the leading causes of death worldwide. In the last decade, T cell-targeted immunotherapy has revolutionized the treatment of patients with extensive solid tumor burden who were previously considered “terminally ill“. A particularly successful example of immunotherapy is immune checkpoint inhibitors (ICI), which reinvigorate the potent anti-tumor efficacy of endogenous T cells.^2^ In general, the response to immunotherapy is dependent on the presence and activation of effector T cells in the tumor microenvironment (TME) and subsequent lysis of cancerous cells. However, the TME of large solid tumors can create physical barriers and block immune cell infiltration. To date, we lack specific drugs that allow us to remodel the TME and allow effective migration of T cells into the tumors.

There is currently great interest in investigating the physical properties of the ECM network as a potential target for immune therapies. The biological and physical characteristics of the TME are decisive for tumor growth but even more so for antitumor immunity and a potential tumor therapy. The role of physical properties such as solid stress, stiffness, fluid pressure, and microarchitecture of the extracellular matrix (ECM) have thus far been underappreciated, but are beginning to gain recognition for the importance of their role in the context of the TME and tumor treatment.^3^ Herein, collagen – as a major component of the ECM–, its structure, density, and degree of mineralization determine the direct interplay with cells, resulting in physical properties ranging from very stiff (bone) to compliant tissues (tendons). Collagen in healthy tissue acts as a guide for T cells to migrate along. Within the TME however, the role of collagen is more complex. In peripheral tumor regions, the collagen network facilitates the migration of activated T cells, whereas towards the center of a tumor mass, the increased density of the collagen matrix hinders T cell migration.^4^

Sun et al.^1^ identify the extracellular domain (ECD) of Discoidin domain receptor 1 (DDR1) as a key component responsible for isotropic collagen fiber alignment, establishing a physical barrier around tumors, thereby preventing T cell invasion. The authors found that DDR1-ECD is thus, in part, responsible for immune cell exclusion and contributing to the failure of antitumor immunity. They demonstrate that breast cancer cell lines that lack DDR1 fail to engraft in immunocompetent mice, while growing at the same rate as wild-type tumors in immunodeficient mice. Analysis of tumor tissue of the immunocompetent mice revealed that DDR1-KO tumors display significantly increased CD8 T cell counts. Bioinformatic analysis of human breast cancer samples confirmed an association between DDR1 expression level and CD8A expression or CD8 T cell signatures in human patients.

DDR1 is a single-pass receptor tyrosine kinase with an ECD which can be shed by matrix metalloproteases and binds collagen. DDR1’s main task is to facilitate the communication of cells with their respective microenvironment, and it is expressed in different developmental phases and in various tissues. It is commonly known to be overexpressed in human tumors. Surprisingly, the tyrosine kinase activity was not responsible for the observed effect of immune exclusion: Screening of different DDR1 mutants revealed that the ECD of DDR1 was solely responsible for decreased CD8 infiltration and that the effect is dependent on collagen-binding. Careful spatial assessment of tumors revealed that DDR1 prevents penetration of CD8 T cells into the tissue core, trapping T cells at the margins of the tumor mass.

To create a novel therapeutic agent to reconfigure the ECM of tumors, Sun and colleagues selected monoclonal antibodies targeting the ECD of human DDR1. The binding of monoclonal antibodies to DDR1-ECD led to collagen fiber rearrangement allowing immune cell migration in vitro (illustrated in Fig. 1). In an immunocompetent mouse model of triple-negative breast cancer, application of anti-huDDR1 antibodies induced complete tumor regression in 7 out of 18 mice providing a promising proof-of-principle of therapeutic efficacy. These results warrant future investigation of the anti-huDDR antibody in combination with other immunotherapies, such as ICI or adoptive transfer of tumor-specific T cells. Herein, challenging preclinical models with large, established tumor masses may be optimal to investigate the synergy of such co-treatments.

**Fig. 1:**
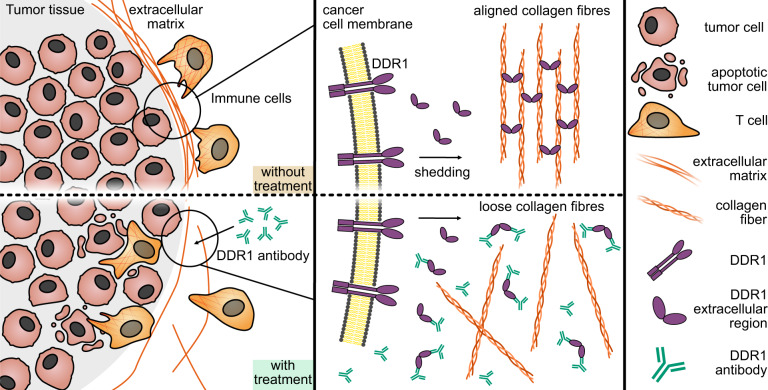
Functional mechanisms of DDR1. (top) DDR1-ECD facilitating collagen fiber alignment and immune cell exclusion. (bottom) Treatment with monoclonal antibody captures DDR1-ECD, preventing collagen fiber alignment, resulting in immune cell invasion and tumor remission

The study suggests that reconfiguration of the ECM, via the effects of anti-huDDR1, in combination with immunotherapy could lead to synergistic anti-tumor effects. In addition, reprogrammed T cells could be pre-conditioned to push beyond these dense barriers to facilitate infiltration. Using T cell pre-exposure to nanoporous physical constraints was shown to boost their activation and proliferation capacity^5^ and might further help to enhance their infiltration capacity. However, there is still a long way to go until the clinical reality of these exciting findings can be realized, with the safety of such an approach being of utmost importance. Due to the ubiquitous role of collagen-cell interactions, unintended effects or toxicity of anti-huDDR1 antibodies will need to be tested in relevant models. Timing, dosage, and length of anti-huDDR1 treatments have to be carefully evaluated in regard to increase of anti-tumor efficacy, but also adverse events. Careful analysis of how anti-huDDR antibody treatment affects other pro-inflammatory or immunosuppressive immune cell subsets locally at and within the tumor, but also at distant sites must be evaluated in syngeneic models. The study additionally lays the groundwork for other basic research studies and DDR1-mediated ECM-immune cell interactions, as immune cells also orchestrate matrix-reconfiguration in other processes, such as wound healing or after bone fracture.

## References

[1] Sun, X. et al. Tumour DDR1 promotes collagen fibre alignment to instigate immune exclusion. *Nature*10.1038/s41586-021-04057-2 (2021). 10.1038/s41586-021-04057-2 PMC8839149 34732895

[2] Robert C (2020). A decade of immune-checkpoint inhibitors in cancer therapy. Nat. Commun..

[3] Nia HT, Munn LL, Jain RK (2020). Physical traits of cancer. Science.

[4] Boissonnas A, Fetler L, Zeelenberg IS, Hugues S, Amigorena S (2007). In vivo imaging of cytotoxic T cell infiltration and elimination of a solid tumor. J. Exp. Med..

[5] Aramesh M (2021). Nanoconfinement of microvilli alters gene expression and boosts T cell activation. Proc. Natl Acad. Sci. USA.

